# Linkage disequilibrium and haplotype block structure in a composite beef cattle breed

**DOI:** 10.1186/1471-2164-15-S7-S6

**Published:** 2014-10-27

**Authors:** Fabiana Barichello Mokry, Marcos Eli Buzanskas, Maurício de Alvarenga Mudadu, Daniela do Amaral Grossi, Roberto Hiroshi Higa, Ricardo Vieira Ventura, Andressa Oliveira de Lima, Mehdi Sargolzaei, Sarah Laguna Conceição Meirelles, Flávio Schramm Schenkel, Marcos Vinicius Gualberto Barbosa da Silva, Simone Cristina Méo Niciura, Maurício Mello de Alencar, Danísio Prado Munari, Luciana Correia de Almeida Regitano

**Affiliations:** 1 Department of Genetics and Evolution, Federal University of São Carlos, Rodovia Washington Luiz, km 235, PO BOX 676, 13565-905, São Carlos, Brazil; 2 Departamento de Ciências Exatas, UNESP - Univ Estadual Paulista, Faculdade de Ciências Agrárias e Veterinárias, Jaboticabal, SP, 14884-900, Brazil; 3 Embrapa Southeast Livestock, Rodovia Washington Luiz, km 234, PO BOX 339, 13560-970, São Carlos, Brazil; 4 Centre for Genetic Improvement of Livestock, University of Guelph, 50 Stone Road East N1G 2W1, Guelph, Ontario, Canada; 5 Embrapa Agricultural Informatics, Avenida André Tosello, 209, PO BOX 6041, 13083-886, Campinas, Brazil; 6 Department of Animal Science, Federal University of Lavras, PO BOX 3037, 37200-00, Lavras, Brazil; 7 Embrapa Dairy Cattle, Rua Eugênio do Nascimento, 610, 36038-330, Juiz de Fora, Brazil

**Keywords:** animal breeding, bovine, Canchim, consistency of gametic phase, single nucleotide polymorphism, persistence of LD phase

## Abstract

**Background:**

The development of linkage disequilibrium (LD) maps and the characterization of haplotype block structure at the population level are useful parameters for guiding genome wide association (GWA) studies, and for understanding the nature of non-linear association between phenotypes and genes. The elucidation of haplotype block structure can reduce the information of several single nucleotide polymorphisms (SNP) into the information of a haplotype block, reducing the number of SNPs in a coherent way for consideration in GWA and genomic selection studies.

**Results:**

The maximum average LD, measured by r^2 ^varied between 0.33 to 0.40 at a distance of < 2.5 kb, and the minimum average values of r^2 ^varied between 0.05 to 0.07 at distances ranging from 400 to 500 kb, clearly showing that the average r^2 ^reduced with the increase in SNP pair distances. The persistence of LD phase showed higher values at shorter genomic distances, decreasing with the increase in physical distance, varying from 0.96 at a distance of < 2.5 kb to 0.66 at a distance from 400 to 500 kb. A total of 78% of all SNPs were clustered into haplotype blocks, covering 1,57 Mb of the total autosomal genome size.

**Conclusions:**

This study presented the first high density linkage disequilibrium map and haplotype block structure for a composite beef cattle population, and indicates that the high density SNP panel over 700 k can be used for genomic selection implementation and GWA studies for Canchim beef cattle.

## Background

With the advance in high-throughput single nucleotide polymorphism (SNP) detection and genotyping technologies, genome-wide association (GWA) and genomic selection (GS) studies in livestock have become of great interest. Nonetheless, both types of studies rely on the extent of linkage disequilibrium between markers across the genome. Linkage disequilibrium (LD) can be defined as the non-random relationship of alleles between two loci within a population. The development of LD maps and the characterization of haplotype block structure at the population level are useful parameters for guiding GWA, candidate gene and candidate region studies [1], as well as for understanding the nature of non-linear association between phenotypes and genes. LD in a population can be affected by some factors, such as population structure, migration, selection, genetic drift, mutations and recombination rates [2].

A variety of studies can be found regarding linkage disequilibrium in cattle populations. The first genome-wide LD study was conducted in a Dutch black-and-white dairy cattle population based on 284 microsatellite markers [3]. From there on, many other studies have been performed and confirmed extensive LD in cattle [4-8], being followed by a second generation of LD maps developed with 30 k or more SNPs spanning the entire bovine genome [9-12]. Lastly, a recent high density LD study on Nellore using 700 k SNPs has been published [13], which can be considered the third generation of LD maps, concluding that the estimated LD for SNPs within a physical genomic distance of 30 kb corroborates the use of the 700 k SNPs panel for genomic selection implementation in Nellore cattle.

Haplotype block studies are not as common as LD map studies in cattle. The elucidation of haplotype block structure can bring important considerations for GWA and GS studies, such as the possibility of selecting a set of SNPs with the prospect of reducing the information of several SNPs into the information of a haplotype block, reducing the number of SNPs in a coherent way [14], and optimizing the design and analysis of GWA. Haplotype blocks can also be used for detection of genomic regions under selection during evolution, and identification of signatures of recent positive selection [15].

With the new releases of commercial high density SNP panels with over 700 k SNPs, it is possible to build high definition LD maps and haplotype blocks. In this study, we used the Illumina BovineHD BeadChip to investigate LD and persistence of LD phase patterns, as well as the haplotype block structure for Canchim, a composite breed of beef cattle. Canchim was originated in the early 1960's in Brazil from crosses between *Bos primigenius indicus *(zebu) and *Bos primigenius taurus *(Charolais) animals [16]. The final composition of Canchim animals is 5/8 parts Charolais and 3/8 parts zebu (currently, the Nellore breed is the most common zebu breed used in the crosses to obtain Canchim animals). However, the Canchim Breeding Association allows for four different crossing schemes to generate Canchim animals, and one of these schemes, called UEPAE (Unidade de Execução de Pesquisa de Âmbito Estadual), produces animals with, on average, 3.1% more Charolais in the final composition, called MA animals, but they are still evaluated with other Canchim animals taking into account the differences in the percentage of each original breed (zebu and Charolais) [17].

## Methods

### Animals

In respect to the Canchim composition particularity, a sample of 399 animals (285 Canchim, 114 MA) was selected, and Canchim animals will be referred to as CA, the MA animals as MA, and the joint group of Canchim and MA animals as CAN. These 399 animals belong to seven herds located in two Brazilian states (São Paulo and Goiás), and are registered in the Canchim Breeding Association database. This study has been performed with the approval of the Embrapa Southeast Livestock Ethical Committee of Animal Use (CEUA-CPPSE) under protocol number 02/2009.

The Canchim population is considered to be rather small, especially when compared to other breeds in Brazil. The herd is estimated to contain approximately 30,000 animals, according to data from the Canchim Breeding Association [18], and supplied just 0.12% of all beef cattle semen sales in Brazil [19]. However, this data set originated from 50 different bulls, comprised of Canchim and MA animals from the breed developer farm and other farms. This is representative of 0.01% of the entire Canchim population. For these reasons, we ignored the probable founder effects, and considered this sample to be representative of the current Canchim population.

### Genotyping and SNP quality control

The 399 animals described above were genotyped using the BovineHD BeadChip (Illumina Inc., San Diego, CA), which consists of 786,799 SNPs evenly distributed along the bovine genome with an average distance of 3 kb. In this study, sex chromosomes and loci without an assigned position in the Cattle Genome Assembly UMD 3.1 [20] were discarded, as well as animals with a call rate < 0.90. The SNP quality control was carried out according to low call rates (< 0.90), Hardy-Weinberg equilibrium (< 0.0001), and minor allele frequency (MAF) < 0.05, as extreme values of MAF can reduce the power to properly estimate linkage disequilibrium and persistence of LD phase [21].

### Linkage disequilibrium and persistence of linkage disequilibrium phase

The data set was divided into Canchim and MA animals for an exploratory analysis of persistence of LD phase between both genetic groups, and LD estimation was performed using the SNPPLD tool available in the gebv software [22]. The LD measurement adopted in this study was r^2 ^[23], which is the correlation coefficient between SNP pairs, and was calculated according to the following equation:

$$r_{ij}^{2}=\frac{p_{ij}-p_{i}\times p_{j}^{2}}{p_{i}\left(1-p_{i}\right)\times p_{j}\left(1-p_{j}\right)}$$

where *p_ij _*is the frequency of the two-marker haplotype, and *p_i_*, and *p_j _*are the marginal allelic frequencies in the *i^th ^*and *j^th ^*SNP, respectively [24]. The value of r^2 ^can vary from 0 to 1, where zero means no correlation between SNP pairs, and 1 means perfect correlation between the SNP pairs. Due to the significant amount of possible SNP pair-wise comparisons, the r^2 ^calculation was limited to SNPs within maximum distances of 500 kb from each other, since r^2^-values obtained using SNPs with distances over 500 kb presented low LD values (data not shown), and to estimate all SNPs pair-wise comparisons would exponentially increase computing processing. Paternal and maternal haplotypes were utilized for the estimation of LD.

The persistence of LD phase was evaluated across genetic groups (CA and MA) by the Pearson correlation of the square root of r^2 ^(*r*), by attributing the appropriate sign based on the calculated D value, called *signed r*. Persistence of LD phase calculation was performed according to the following equation:

$$D=p_{ij}-\left(p_{i}\times p_{j}\right)$$

where *p_ij_, p_i_*, and *p_j _*were defined as stated above. The results were ordered by chromosome (chr) and distance between SNPs.

### Haplotype block structure

Haplotype block refers to a combination of alleles linked along a chromosome, which are inherited together from a common ancestor [25]. The haplotype block structure study was carried out using the joint CAN group. For doing this, the same quality control filters were applied through the PLINK v1.07 software [26], and the phase and haplotype reconstruction were performed using the BEAGLE Version 3.3.1 software [27] for each chromosome. Afterwards, the Haploview [28] software, which uses haplotype block definition by Gabriel et al. [29] by default, was used for estimating haplotype block patterns for the 29 pairs of autosome chromosomes, within SNPs at a maximum distance of 500 kb.

## Results

**Figure 1:**
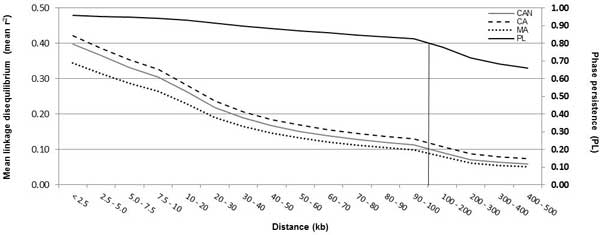
**Average r^2 ^values for CA, MA and CAN animals, and persistence of LD phase (PL) between CA and MA animals with respect to physical genomic distance (kb)**.

**Figure 2:**
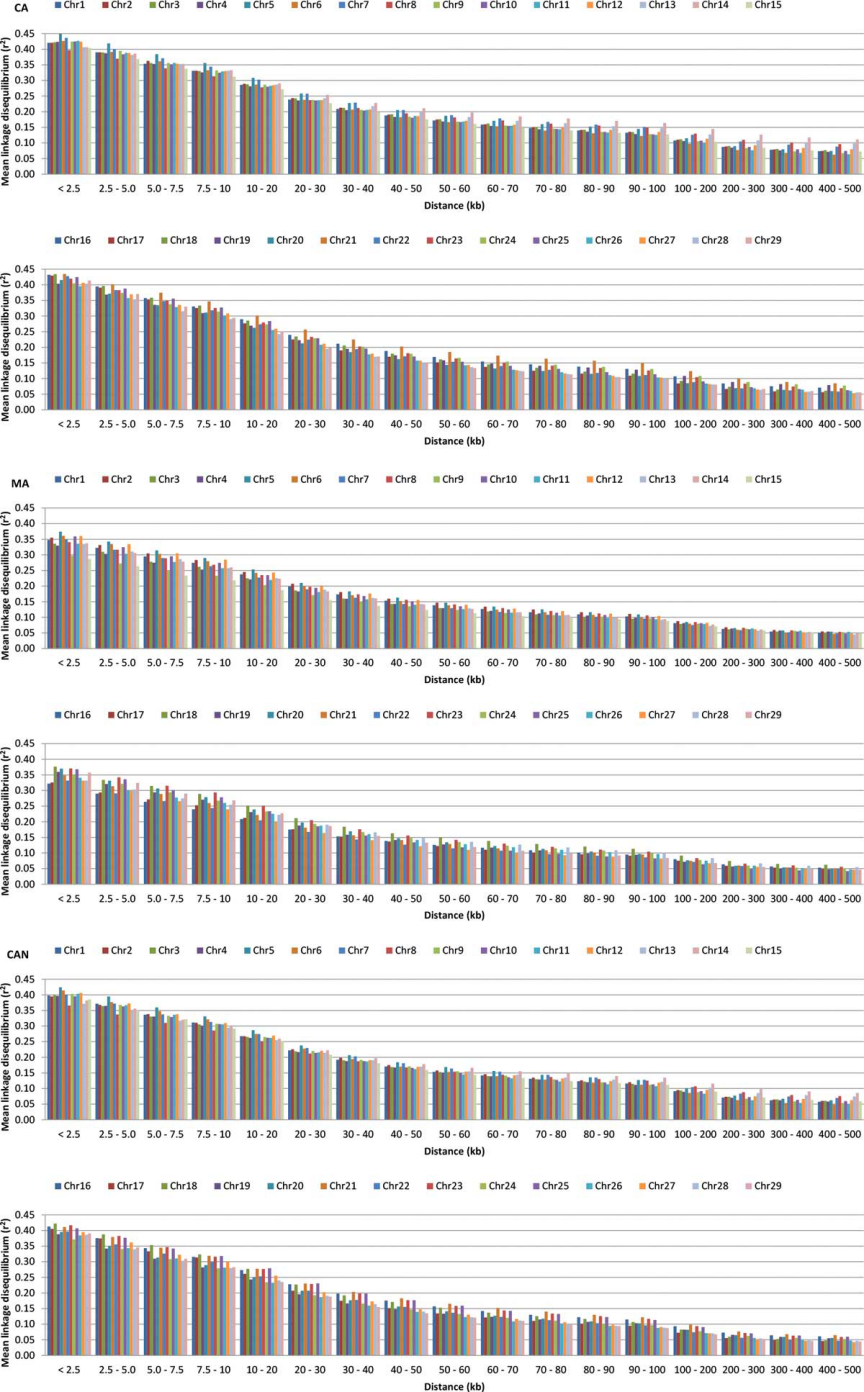
**Mean values of linkage disequilibrium (r^2^) per chromosome (chr:1-29) according to distance (in kb) between markers for CA, MA, and CAN animals**.

A total of 395 animals (283 CA and 112 MA) passed the quality control filters from the SNPPLD software, yielding a total of 716,089 SNPs for CA, 658,132 SNPs for MA, and 713,615 SNPs for CAN animals. The number of SNP pairs showed small variation among CA, MA, and CAN due to the quality control filtering, in which some SNPs were included for CA, MA, and CAN, and some other SNPs were not included, as shown in Table 1. The maximum average r^2 ^of 0.40 was obtained for CA animals at a distance of < 2.5 kb, while MA animals obtained an average r^2 ^of 0.33, and the joint population (CAN) resulted in an average r^2 ^of 0.38 (Table 1). The average minimum values of r^2 ^(0.07 CA, 0.05 MA, and 0.06 CAN) were obtained at a distance from 400 to 500 kb (Table 1), clearly showing that the average r^2 ^reduced with the increase in SNP pair distances. The average r^2^, up to the physical genomic distance of 500 kb, was slightly higher for CA animals, followed by CAN, and lastly, by MA animals (Figure 1). The persistence of LD phase between CA and MA animals showed higher values at shorter genomic distances, decreasing with the increase in physical distance (Figure 1). The persistence of LD phase varied from 0.96 at distances < 2.5 kb, to 0.66 at distances between 400 and 500 kb, with an overall average of 0.85 (Table 1). Even though the LD decay with the increase in distance is clear (Figure 1), it is possible to identify some chromosomes (chrs) with lower LD decay among genetic groups, as in chrs 5, 14, and 21 (Figure 2).

**Table 1:** Summary of SNP pairs, average linkage disequilibrium (r^2^), standard deviation (SD), median, persistence of LD phase (PL) between CA and MA genetic groups by genomic distance.

Distance (kb)	CA^1^				MA^2^				CAN^3^				PL
				
	SNP pairs	r^2^	SD	Median	SNP pairs	r^2^	SD	Median	SNP pairs	r^2^	SD	Median	
< 2.5	407,763	0.40	0.38	0.27	401,469	0.33	0.33	0.21	409,164	0.38	0.36	0.26	0.96
2.5 - 5.0	561,174	0.37	0.37	0.23	552,492	0.30	0.32	0.18	563,114	0.34	0.35	0.22	0.95
5.0 - 7.5	531,897	0.34	0.35	0.20	523,596	0.27	0.31	0.15	533,663	0.31	0.33	0.18	0.95
7.5 - 10	519,006	0.31	0.34	0.18	510,908	0.25	0.29	0.13	520,759	0.29	0.32	0.16	0.94
10 - 20	2,007,682	0.27	0.32	0.14	1,976,524	0.22	0.27	0.10	2,014,438	0.25	0.29	0.13	0.93
20 - 30	1,946,916	0.23	0.29	0.11	1,915,942	0.18	0.24	0.08	1,953,735	0.21	0.26	0.10	0.91
30 - 40	1,912,997	0.20	0.26	0.09	1,882,863	0.16	0.22	0.07	1,919,643	0.18	0.24	0.08	0.90
40 - 50	1,893,506	0.18	0.25	0.08	1,863,542	0.14	0.20	0.06	1,900,075	0.16	0.22	0.07	0.88
50 - 60	1,878,923	0.16	0.23	0.07	1,848,963	0.13	0.19	0.05	1,885,467	0.15	0.21	0.06	0.87
60 - 70	1,866,531	0.15	0.22	0.06	1,836,777	0.11	0.18	0.05	1,873,058	0.13	0.20	0.06	0.86
70 - 80	1,857,712	0.14	0.21	0.06	1,828,186	0.11	0.17	0.04	1,864,285	0.13	0.19	0.05	0.85
80 - 90	1,852,950	0.13	0.20	0.06	1,823,709	0.10	0.16	0.04	1,859,564	0.12	0.18	0.05	0.84
90 - 100	1,848,344	0.13	0.19	0.05	1,819,128	0.09	0.15	0.04	1,855,025	0.11	0.17	0.05	0.83
100 - 200	18,280,956	0.10	0.17	0.04	17,988,347	0.07	0.12	0.03	18,345,281	0.09	0.14	0.04	0.78
200 - 300	18,079,858	0.08	0.14	0.03	17,787,703	0.06	0.09	0.03	18,143,163	0.07	0.11	0.03	0.72
300 - 400	17,964,483	0.07	0.13	0.03	17,674,588	0.05	0.08	0.02	18,027,967	0.06	0.10	0.02	0.68
400 - 500	17,862,211	0.07	0.12	0.03	17,575,556	0.05	0.07	0.02	17,926,215	0.06	0.09	0.02	0.66

1CA=Canchim animals; 2MA=MA genetic group animals; 3CAN= CA and MA animals joined together

**Figure 3:**
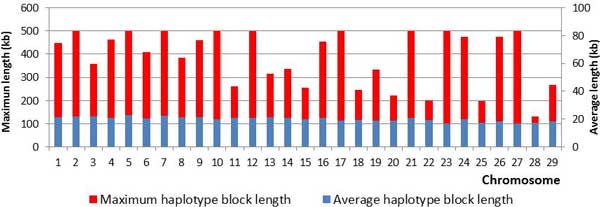
**Maximum and average haplotype block length per chromosome**.

**Figure 4:**
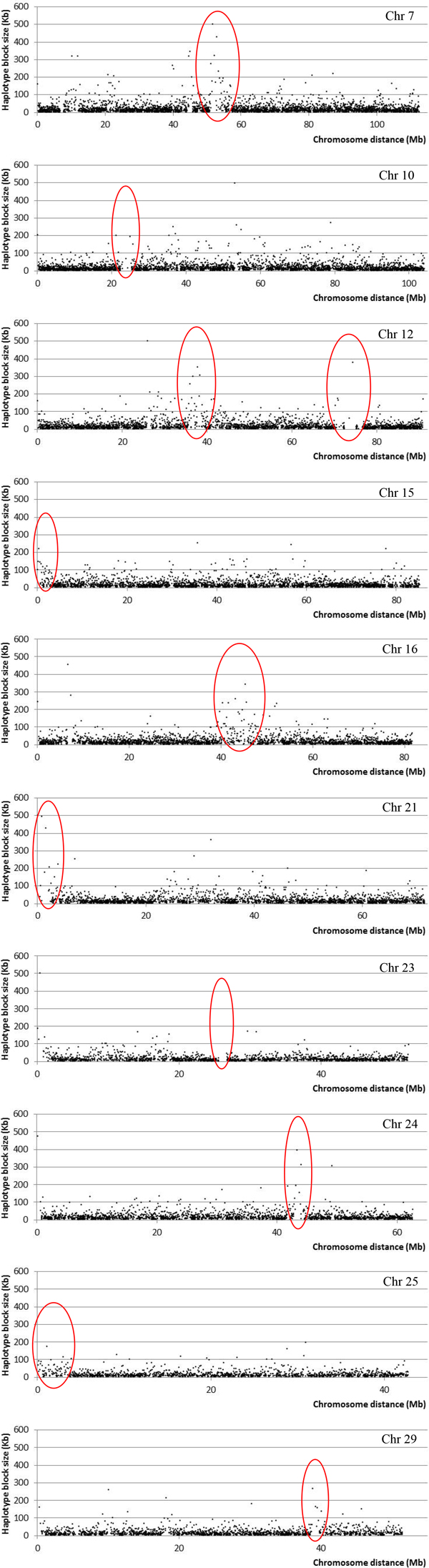
**Haplotype block distributions for chromosomes (chrs) 7, 10, 12, 15, 16, 21, 23, 24, 25 and 29 (red ellipses for highlighted area of uncommon haplotype block distribution: gaps without any haplotype - chrs10, 12, 23; areas with higher frequencies of larger haplotypes - chrs 7, 12, 16, 24, 29; and uncommon haplotype pattern distributions at the extreme ends - chrs 15, 21, 25)**.

The quality control filtering for the haplotype block study was performed using the PLINK v1.07 software [26], which permitted 664,027 SNPs to remain in this study, with an average distance between SNP markers of 3.8 kb, leading to a total autosomal genome size of 2.51 Mb. Haplotype blocks formed by only two SNPs were discarded to avoid spurious block formation. A descriptive summary of the haplotype block analysis can also be found in Table 2. From the previous SNPs, 517,393 were clustered into haplotype blocks, which correspond to 78% of all SNPs, covering 1.57 Mb of the total autosomal genome size (Table 2). Chr 1 showed the highest number of SNPs (41,830) and haplotype blocks (4,787), while chr 25 presented the smallest number of SNPs (11,671) and haplotype blocks (1,396) (Table 2), with an overall average haplotype block length of 20 kb (Figure 3). Overall, 61% of chromosome lengths were covered by haplotype blocks, with chrs 23 and 28 showing the smallest coverage (53%), while chrs 2, 7 and 9 showed the greatest coverage (66%) (Table 2). Figure 4 displays the haplotype distributions on chrs 7, 10, 12, 15, 16, 21, 23, 24, 25, and 29 with some uncommon haplotype distributions (gaps without any haplotype - chrs 10, 12, 23; areas with higher frequencies of larger haplotypes - chrs 7, 12, 16, 24, 29; and uncommon haplotype pattern distributions at the extreme ends - chrs 15, 21, 25).

**Table 2:** Haplotype block summary per chromosome (chr).

Chr	Number of SNPs	Chr length (Mb)	Average SNPs distance (kb)	Number of blocks	Block coverage length (Mb)	% Chr block coverage	SNPs in blocks	% SNPs in blocks
1	41,830	158,306	3.78	4,787	103,419.90	65	33,411	80
2	36,178	136,763	3.78	4,118	90,048.32	66	28,981	80
3	32,061	121,405	3.79	3,653	79,147.48	65	25,442	79
4	31,800	120,635	3.79	3,677	76,877.07	64	25,018	79
5	31,160	121,182	3.89	3,446	79,131.14	65	25,222	81
6	32,480	119,423	3.68	3,764	76,983.56	64	25,998	80
7	29,879	112,595	3.77	3,310	74,287.94	66	23,993	80
8	29,907	113,346	3.79	3,414	72,761.84	64	23,790	80
9	28,552	105,667	3.70	3,275	69,607.56	66	22,817	80
10	27,824	104,283	3.75	3,211	64,087.69	61	21,370	77
11	28,788	107,269	3.73	3,252	68,632.31	64	22,857	79
12	23,562	91,125	3.87	2,665	55,775.42	61	18,327	78
13	20,886	84,207	4.03	2,461	52,981.54	63	16,071	77
14	22,235	84,035	3.78	2,596	54,099.85	64	17,579	79
15	22,019	85,229	3.87	2,639	52,643.91	62	17,107	78
16	22,021	81,685	3.71	2,495	51,656.07	63	17,177	78
17	20,144	75,146	3.73	2,377	45,306.63	60	15,517	77
18	17,611	65,913	3.74	2,032	39,490.48	60	13,492	77
19	17,032	63,964	3.76	1,982	37,624.33	59	12,606	74
20	19,407	71,950	3.71	2,324	43,679.74	61	15,038	77
21	19,101	71,568	3.75	2,205	46,122.94	64	14,919	78
22	16,610	61,279	3.69	1,893	36,982.78	60	12,655	76
23	13,695	52,459	3.83	1,660	27,629.29	53	9,482	69
24	16,983	62,542	3.68	1,948	38,866.11	62	13,065	77
25	11,671	42,823	3.67	1,396	23,924.44	56	8,522	73
26	13,820	51,642	3.74	1,657	30,037.78	58	10,402	75
27	11,822	45,400	3.84	1,447	24,658.49	54	8,573	73
28	11,872	46,243	3.90	1,411	24,333.07	53	8,406	71
29	13,077	51,180	3.91	1,578	28,700.01	56	9,556	73
**Total**	664,027	2,509,262	3.78 ± 0.08	76,673	1,569,498	61 ± 4	517,393	77 ± 3

## Discussion

The choice of using r^2 ^instead of D' for assessing LD measurements was due to the fact that it is less affected by allele frequencies in a finite population size compared to D', which tends to overestimate LD in small samples and low frequency alleles [12,30,31]. According to the literature, mean r^2^-values above 0.30 can be considered a strong LD and useful for QTL mapping [31], while an mean r^2^-value of 0.20 is considered enough to achieve an accuracy of 0.85 for genomic breeding value (GBV) estimation [32]. Mean values of r^2^=0.30 and above were found in CA animals extending to the physical genomic distance of 10 kb, being followed by CAN animals at a distance of 7.5 kb, and lastly by MA animals to the physical genomic distance of 5 kb (Table 1). Considering the great appeal of using SNPs for GBV estimation and the above mentioned threshold of r^2^=0.20, for CA animals GBV can be estimated by using SNP markers at a distance between 30-40 kb, for CAN animals by using SNP markers at distances between 20-30 kb, and for MA animals by using SNP markers at distances between 10-20 kb. Even though making comparisons between LD studies is difficult, as the level of LD varies due to sample size, marker types, density, and population history [33], the results obtained in this study are in agreement with a recent LD study using approximately 700 kb SNPs in Nellore cattle, which reports levels of LD (r^2^) higher than 0.30 for SNP markers spanning to a physical genomic distance of 3 kb, and an r^2 ^higher than 0.20 for SNP markers continuing to a physical genomic distance of 20 kb [13].

Animals from the MA genetic group showed lower levels of LD compared to CA animals (Table 1). This might be explained in part by the crossing system used to obtain MA animals (CA × zebu = F1, and F1 × Charolais = MA), while most of the CA animals are obtained by CA × CA crosses. Some studies have shown that the average LD decay with the increase in physical genomic distance between loci is more accentuated in crossbred and admixed populations compared to purebred populations [34,35]. One of the reasons is that individuals from crossbred populations are less related to each other (further common ancestor), leading to LD haplotypes in crossbred populations being narrower than LD haplotypes in purebreds [35]. In the MA situation, these animals are obtained by initially crossing Canchim with zebu animals, and the progeny are then crossed with Charolais animals. Despite the decrease of LD levels along with the increase in physical genomic distance, the LD behavior also showed variability among chromosomes and chromosomal regions. These variations can be attributed to many factors, such as differences in recombination rates between and within chromosomes, heterozygosity, selection effects, and genetic drift.

The understanding of the persistence of LD phase is essential for effective genomic selection across admixed populations or crossbred animals, as a pair of SNPs can exhibit the same value of r^2 ^between two populations, but in different LD phases [36]. The correlation of the *signed r *value represents the degree of genetic relationship between populations [37], and between MA and CA animals it was over 0.80 continuing to a genomic distance of 100 kb between both genetic groups (Table 1), and decreased to a minimum of 0.66 extending to a genomic distance of 500 kb. According to previous studies [35,37], high correlation values imply in consistency of LD phase, and considering a threshold of r^2 ^= 0.20 to achieve an accuracy of 0.85 for genomic breeding value estimation [32], the persistence of LD phase between MA and CA animals stayed over 0.91 until reaching a genomic distance of 30 kb, meaning that one population can be used to predict the performance of the other (e.g. MA animals) [12], which allows for considering both genetic groups (CA and MA animals) together as one breed (CAN animals) for future studies and for genetic evaluation purposes.

There are many published studies on LD and haplotype block properties for cattle, which vary in many aspects (breed of interest, marker types, marker density, and chromosome regions), yielding average haplotype block sizes from a few kb in length (5.7 kb considering 2 or more SNPs [9], 26.2 kb considering 4 or more SNPs [8]) to hundreds of kb in length (700 kb [6]), and covering from 2.18% to 4.67% [4,10] of the detected genome. However, these studies used smaller marker densities, with an average distance of 62 kb between adjacent markers [10]. Another study, which considered only high-density markers in specific areas of the bovine genome (approximate distances of 5 kb between adjacent markers), reported an average block size of 10.3 kb across many breeds, an average of 3.8 SNPs per block, and a total of 34% of the high-density areas covered by haplotype blocks [9]. These values are still smaller than the ones reported in this study for percentage of covered genome (61%), average number of SNPs per block (6.64 SNPs), and a total of 78% SNPs in haplotype blocks, corroborating the assumption that as the marker density increases, the more haplotype blocks of smaller sizes will be identified. On the other hand, this is not supported in total by a study in German Holstein cattle, which did not report relevant variation on haplotype block number with the increase of marker density, but an increase in block coverage percentage [10].

There are many aspects reported in the literature involved in LD shaping, which in turn affects the haplotype block structure, such as meiotic recombination, natural and artificial selection, population bottle necks, genetic drift, and admixture [29,38-42]. However, most of these studies were carried out in humans [39-42], which, among other differences, are not affected by artificial selection and have a higher effective population size than cattle [43]. All these factors play important roles in the haplotype block structure which could be the cause of some of the uncommon haplotype distributions found in Figure 4.

## Conclusions

This study describes the first high-density linkage disequilibrium map and haplotype block structure for a composite beef cattle population. Considering an r^2 ^≥ 0.20 as being useful for genomic breeding value estimations, the results demonstrate that the high density SNP panel used here can be implemented for genomic selection of Canchim beef cattle. The persistence of LD phase between CA and MA animals was consistent, which supports the decision of considering both genetic groups together in future studies and in genetic evaluation programs. Further studies on factors affecting the uncommon haplotype block distribution still need to be carried out in order to better understand the way these factors are shaping the LD and haplotype blocks.

## Availability of supporting data

The genomic data and further supporting data are available upon request from Dr. Luciana Correia de Almeida Regitano (Embrapa Southeast Livestock. Address: Rodovia Washington Luiz, km 234, São Carlos, São Paulo, 13560-970, Brazil; e-mail: luciana.regitano@embrapa.br; telephone: +55 16 34115600).

## Competing interests

The authors declare that they have no competing interests.

## Authors' contributions

FBM, MEB: contributed equally for the development of this research with data analysis, interpretation, figure compositions, manuscript writing and revision. MAM: data analysis, manuscript discussion and revision. DAG: data analysis, interpretation of results, manuscript revision. RHH: data analysis, manuscript discussion and revision. RVV: data analysis, interpretation. AOL: data analysis, interpretation of results, manuscript revision. MS: provided the SNPPLD software, interpretation. SLCM: experimental design, manuscript revision. FSS: results interpretation and discussion. MVGBS: experimental design, manuscript revision. SCMN: experimental design, DNA handling, manuscript revision. MMA: experimental design. DPM: data interpretation. LCAR: experimental design, interpretation, and manuscript revision.
